# Automatic epileptic seizure detection based on EEG using a moth-flame optimization of one-dimensional convolutional neural networks

**DOI:** 10.3389/fnins.2023.1291608

**Published:** 2023-12-14

**Authors:** Baozeng Wang, Xingyi Yang, Siwei Li, Wenbo Wang, Yichen Ouyang, Jin Zhou, Changyong Wang

**Affiliations:** ^1^Beijing Institute of Basic Medical Sciences, Beijing, China; ^2^State Key Laboratory of Intelligent Manufacturing System Technology, Beijing Institute of Electronic System Engineering, Beijing, China; ^3^Chinese Institute for Brain Research, Beijing, China

**Keywords:** moth-flame optimization, convolutional neural networks, hyperparameter optimization, electroencephalogram, epileptic seizure detection

## Abstract

**Introduction:**

Frequent epileptic seizures can cause irreversible damage to the brains of patients. A potential therapeutic approach is to detect epileptic seizures early and provide artificial intervention to the patient. Currently, extracting electroencephalogram (EEG) features to detect epileptic seizures often requires tedious methods or the repeated adjustment of neural network hyperparameters, which can be time- consuming and demanding for researchers.

**Methods:**

This study proposes an automatic detection model for an EEG based on moth-flame optimization (MFO) optimized one-dimensional convolutional neural networks (1D-CNN). First, according to the characteristics and need for early epileptic seizure detection, a data augmentation method for dividing an EEG into small samples is proposed. Second, the hyperparameters are tuned based on MFO and trained for an EEG. Finally, the softmax classifier is used to output EEG classification from a small-sample and single channel.

**Results:**

The proposed model is evaluated with the Bonn EEG dataset, which verifies the feasibility of EEG classification problems that involve up to five classes, including healthy, preictal, and ictal EEG from various brain regions and individuals.

**Discussion:**

Compared with existing advanced optimization algorithms, such as particle swarm optimization, genetic algorithm, and grey wolf optimizer, the superiority of the proposed model is further verified. The proposed model can be implemented into an automatic epileptic seizure detection system to detect seizures in clinical applications.

## 1 Introduction

According to the World Health Organization (2023), ~50 million people of all ages suffer from daily or weekly seizures worldwide, making it one of the most common neurological disorders. Epilepsy is a disorder caused by brain injury or abnormal discharges of neurons in the brain. Its characteristic is the repeated occurrence of transient brain dysfunction, mainly manifested as motor impairment, sensory impairment, or impaired consciousness, which can lead to limb convulsions, confusion, and even life-threatening situations (Bhattacharyya et al., 2017; Kong et al., 2022). The suddenness of epileptic seizures, as well as their self-sustained discharges lasting from a few minutes to several hours, greatly increases the difficulty of detecting them. Therefore, it is clinically important to detect seizures early and intervene to reduce greater suffering for patients (Islam et al., 2022).

In general, in addition to computed tomography, single-photon emission computed tomography, and positron emission tomography, the convenient and fast conventional EEG remains the main means for detecting epileptic seizures (Nikodijevic et al., 2016). In 1964, the International League Against Epilepsy proposed a classification scheme for epileptic seizures for the first time (Caveness et al., 1964). According to the EEG of epileptic patients at different stages, it is clear that EEG features and clinical manifestations have equal diagnostic significance. To overcome the limitations of traditional epileptic seizure detection methods, the automatic detection of seizure type based on an EEG has become a hot research topic in the industry (Zhang Y. et al., 2022). In early clinical testing, epileptic seizure detection and analysis mainly rely on the visual observation and manual annotation of clinicians with specialized knowledge. This process is not only prone to omissions or errors but also increases the burden on doctors. Additionally, it has the disadvantage of relying on physician experience, individual subjectivity, and randomness to detect the presence or absence of epilepsy (Jing et al., 2021). Therefore, it is of great significance to seek automatic, efficient, and objective methods to classify multi-type epileptic EEGs.

Numerous researchers have conducted studies on the automatic detection of epileptic seizures based on an EEG. In the traditional detection methods of epileptic seizure, feature extraction of an EEG is performed using time-domain, frequency-domain, and time-frequency methods, which achieve good results (Hernández et al., 2018). However, these methods require domain expertise and complex EEG feature extraction tasks. Although the recognition model is relatively simple, it has a low recognition rate and poor generalization ability (Kurdthongmee, 2020). With the rapid development of deep learning technology, it is increasingly being applied in the field of brain science, such as neural signal recognition (Zhang H. et al., 2022), EEG classification (Li et al., 2022), and seizure detection (Hernández et al., 2018). In recent years, a plethora of deep learning algorithms, such as convolutional neural networks (CNN) (Sallam et al., 2018), artificial neural networks (Emami et al., 2019), recurrent neural networks (Bongiorni and Balbinot, 2020), long- and short-term memory artificial neural networks (LSTM) (Tsiouris et al., 2018), have received increasing attention and achieved encouraging results in epileptic seizure detection for two-class, three-class, four-class, and five-class classification problems (Zhao et al., 2020). In particular, the time-delay networks model proposed by Alexander Waibel et al. and the first one-dimensional CNN (1D-CNN) were successfully applied to speech recognition (Waibel, 1989). Subsequently, lots of 1D-CNN models have been applied to the research work of sequence models and achieved better recognition results for detecting epileptic seizures from an EEG (Wang et al., 2021; Ra et al., 2023). However, the performance of these deep neural network models directly depends on their hyperparameters (Aliyu and Lim, 2023; Lebal et al., 2023). In practice, the training models require the configuration of several, or even dozens, of network parameters (e.g., number of layers, number of cells, activation function, kernel size, and learning rate) (Kwasigroch et al., 2018; Chetana et al., 2023); the adjustment of these hyperparameters is extremely complex, requiring high-level technical expertise from the designers, and can be tedious and time-consuming. In addition, the optimization cost is high, and repeating the adjustment of hyperparameters through experiments is both inefficient and incomplete, often resulting in unsatisfactory results (Irmak, 2020). Thus, ensuring that the deep neural network framework adapts to a specific dataset and achieves optimal generalization remains one of the important tasks in optimizing the hyperparameters.

Although deep learning models can achieve better epilepsy recognition, no model can optimally adapt to all datasets. However, the optimization of deep learning models has posed new challenges in the field of model hyperparameter tuning (Hoang and Kang, 2019), and scholars from various countries have conducted extensive research. The recognition rate is stable and robust based on time-invariant features extracted from a single-channel EEG using two CNNs for patients with seizures (Zhao and Wang, 2020). The particle swarm optimization algorithm (PSO) is used to adaptively optimize the parameters of the CNN model, and the PSO-CNN model is built to improve the recognition rate of epileptic seizure detection (Lv et al., 2022). Based on the confidence function defined by the complex normal distribution, a new PSO variant named cPSO-CNN is proposed to determine the hyperparameter configuration of CNN (Wang et al., 2019). Similarly, the proposed neural network optimization method has a better performance by optimizing CNN hyperparameters using the genetic algorithm (GA) (Fatyanosa and Aritsugi, 2020). The generalized CNN extracts the most relevant features that can be interpreted and processed based on the Grey Wolf Algorithm (GWO). It has achieved the detection of an abnormal EEG associated with epilepsy, thereby improving the classification accuracy (Thanuja et al., 2023). Usually, numerous hyperparameters can produce a better performance. However, the PSO evaluates and tests the efficiency of forming models with different parameter combinations, which is not only inefficient and time-consuming but also easily falls into the local optimum (Mezzah and Tari, 2023). Therefore, it is necessary to investigate an efficient search solution that can quickly and effectively find an optimal hyperparameter combination (Kim et al., 2020). The moth-flame optimization algorithm (MFO) is a novel meta-heuristic swarm intelligence method that has good global optimization ability and faster convergence speed and optimality seeking as well as few parameters and a simple structure. It has received extensive attention from scholars all over the world (Mirjalili, 2015) and has been successfully applied to many optimization problems, such as scheduling (Elsakaan et al., 2018), parameter estimation (Hazir et al., 2018), and classification (Zawbaa et al., 2016; Shehab et al., 2020). The effectiveness of this optimization algorithm in solving different complex problems in a reasonable time has been demonstrated (Khurma et al., 2020). So far, no effective method has been found to optimize the hyperparameters of 1D-CNN for seizure detection based on MFO. Therefore, the aforementioned research results motivated us to propose a new method for automatically optimizing 1D-CNN hyperparameters based on MFO, without manually adjusting the network structure and hyperparameters.

The main motivation behind the proposed MFO is to find the optimal combination of hyperparameters without manually adjusting the hyperparameters of the network structure. The MFO is then applied to existing CNN models to adjust the hyperparameters. This enables the automatic detection of seizures in a small-sample single-channel EEG and improves the accuracy and universality of the seizure detection system.

## 2 Materials and methods

### 2.1 Epileptic seizure detection system based on 1D-CNN

**Figure 1:**
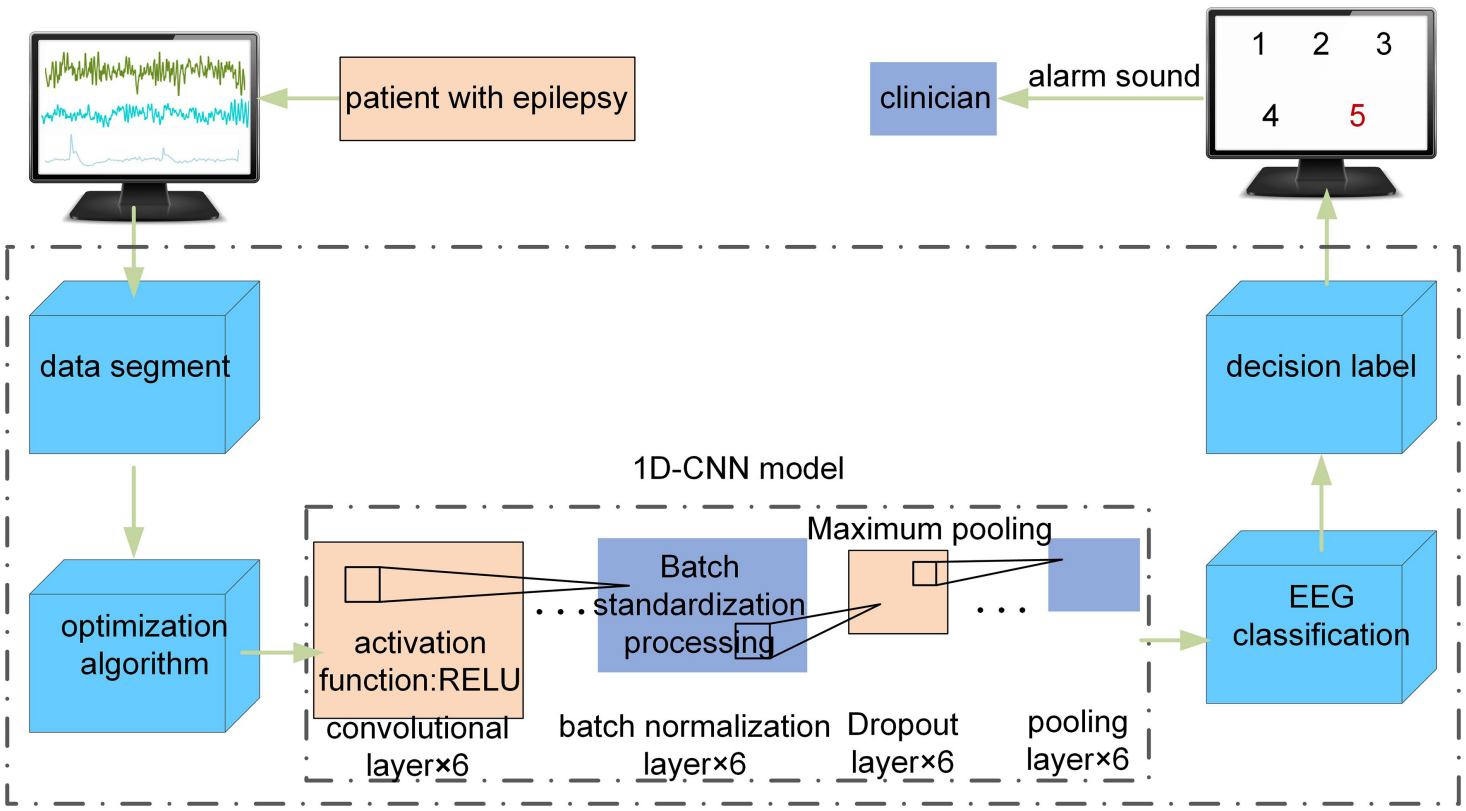
The composition diagram of the epileptic seizure detection system based on an optimized 1D-CNN in clinical applications.

The composition diagram of an epileptic seizure detection system based on the optimized 1D-CNN neural network detector in clinical applications is shown in Figure 1. The system consists of four main modules: (1) the EEG input module, which divides the input EEG into signal segments with a fixed size; (2) the optimization module, which optimizes the 1D-CNN model using the MFO; (3) the EEG feature extraction module, which passes the intercepted signal segments to the optimized 1D-CNN model and extracts EEG features; and (4) the fusion decision module, which inputs the EEG feature matrix to the softmax classifier layer, obtains the corresponding types of the input EEG, and presents the detection result to the doctor or issues an alarm.

The core part of the epileptic seizure detection system is the optimization algorithm in Figure 1. First, the MFO is used to automatically optimize the hyperparameters of the 1D-CNN model. Second, the proposed model is used to automatically classify the EEG time series of these five classes.

### 2.2 Dataset and data partitioning

#### 2.2.1 Description of the EEG dataset

The available EEG dataset produced by the University of Bonn has the characteristics of diversity, large scale, high openness, and widespread use (Andrzejak et al., 2001). This database consists of five different subsets (*Z*, *O*, *N*, *F*, and *S*) denoted by *A*~*E*, respectively. The subsets *A* and *B* are collected using the international 10 − 20 system from five healthy individuals in an awake state with eyes open and eyes closed, respectively. The datasets *C*, *D*, and *E* are derived from EEG archives of presurgical diagnoses of five patients. The datasets *C* and *D* are acquired separately from the hippocampal structure of the opposite hemisphere and within the epileptogenic zone during seizure-free intervals. Then, dataset *E* contains EEG activity during seizure from five patients (Xu et al., 2020).

Each subset contains 100 single-channel EEG segments with a duration of 23.6 s, and each segment has 4, 097 samples with a sampling frequency of 173.61 Hz. These subsets are collected through a 128-channel amplifier system using an average standard reference. A bandpass filter is utilized to remove the noise and artifacts, with low and high cutoff frequencies of 0.53 and 40 Hz respectively set.

**Figure 2:**
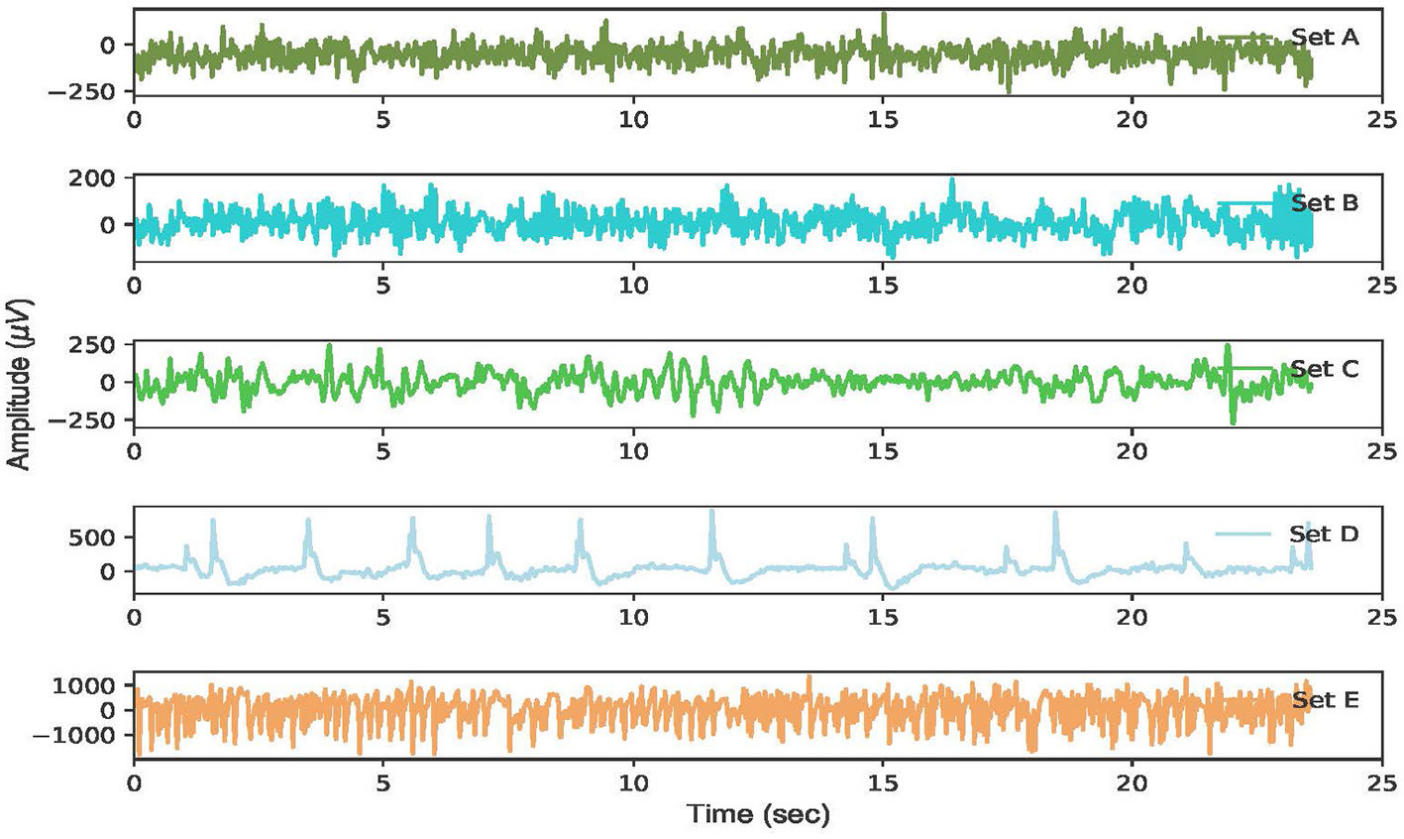
The raw EEG signal waveform corresponding to each of the five datasets of the Bonn dataset.

To illustrate these datasets, the EEG time series of these five classes corresponding to a certain channel is depicted in Figure 2.

The EEG characteristics corresponding to each EEG subset can be observed. The EEG waveforms are almost identical in datasets *A* and *B* from five healthy individuals. Similarly, the EEG amplitudes between datasets *C* and *D* are not substantially different, which increases the difficulty in classifying the EEG. By contrast, the EEG voltages in dataset *E* exceed 1, 000 μV during the ictal phase, which is considerably higher than that of the other EEG datasets.

#### 2.2.2 EEG data augmentation

**Figure 3:**
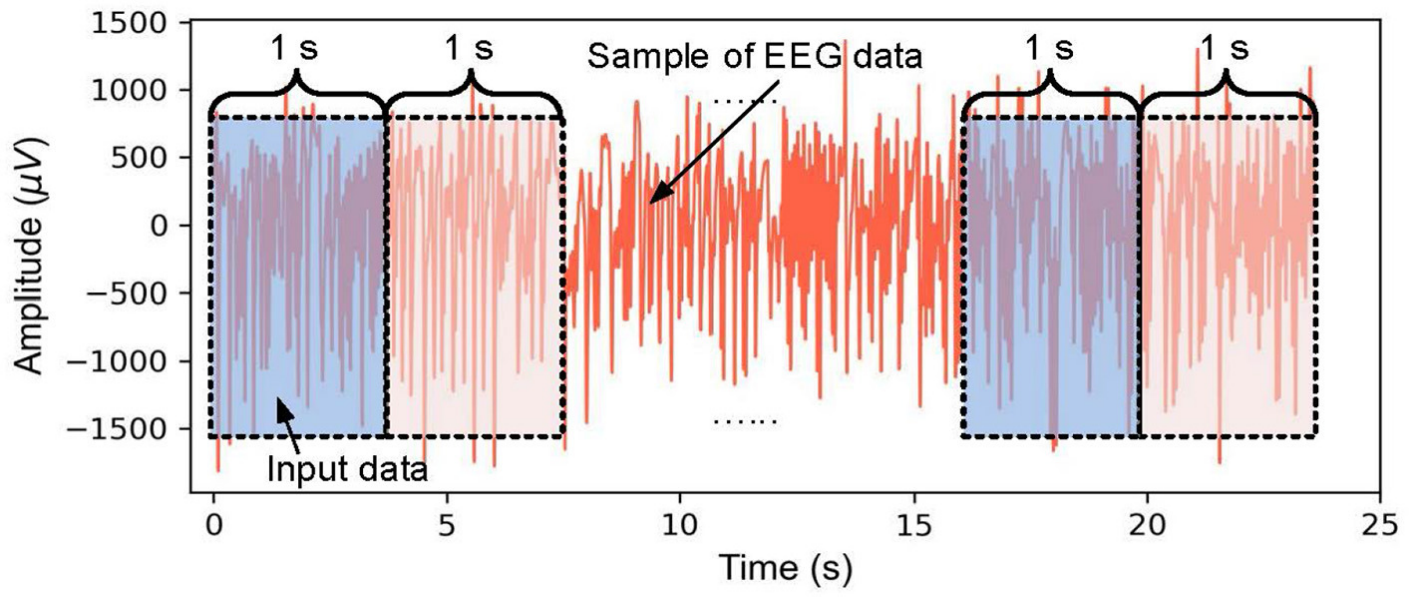
Schematic diagram of EEG data augmentation based on a fixed sliding window.

To extract effective EEG features from a smaller sample, a fixed sliding window is used to divide the EEG time series into segments (Zhang et al., 2017; Ullah et al., 2018). It is important to consider that the data augmentation not only shortens the duration of the EEG segment but also enables timely intervention for epileptic seizures. Additionally, it reduces data redundancy and computational load, making data processing more efficient. This study proposes a sliding window technique with a fixed sliding window of 1 s, as shown in Figure 3.

To increase the number of EEG samples, a single-channel EEG segment of each class is divided every 4, 097 data points into 23 chunks, and the 2, 300 chunks of EEG are obtained for each EEG subset. So a total of 11, 500 samples from five classes are used to evaluate the performance of the proposed method.

### 2.3 Building the MFO-1D-CNN model

#### 2.3.1 Building a 1D-CNN architecture

**Figure 4:**
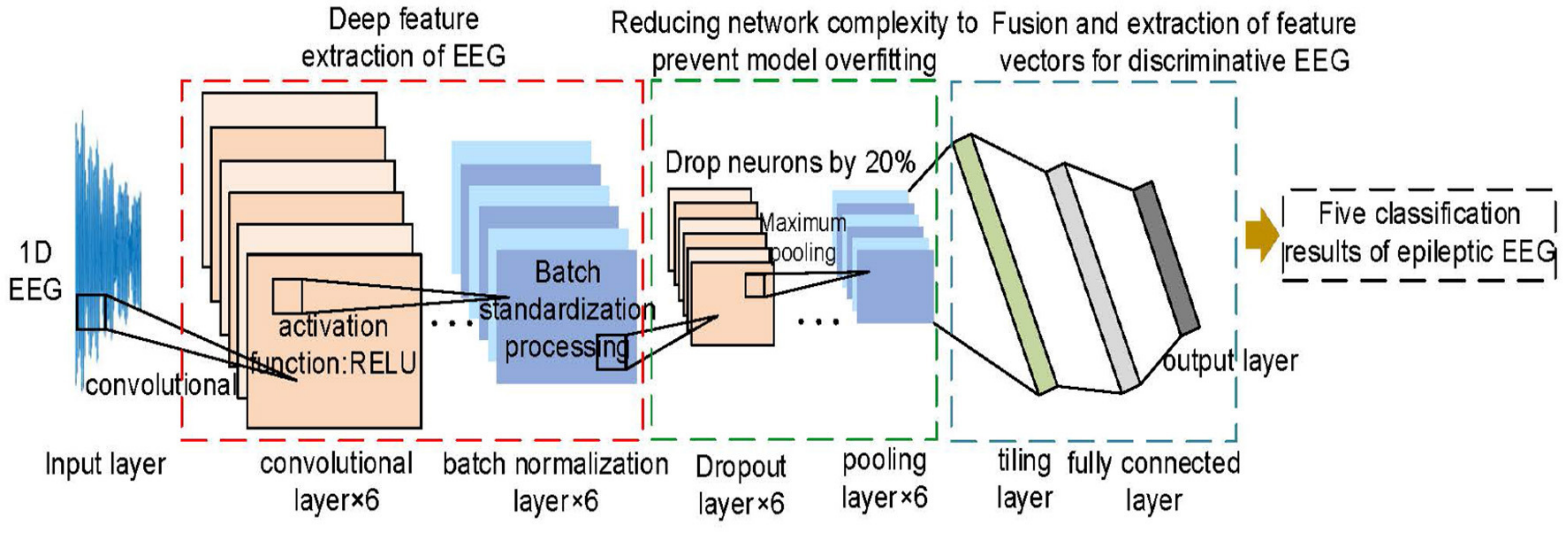
Schematic diagram of the 1D-CNN model for detecting EEG.

Owing to the fact that an EEG is time-series data, a 1D-CNN model is selected for detecting the EEG to improve the accuracy and generalization ability. This network includes the input layer, convolutional layer, pooling layer, dropout layer, and fully connected layer. Unlike the 2D-CNN model, the 1D-CNN model has the advantages of few parameters, easy training, and low computational effort, as shown in Figure 4.

First, it provides alternating layers of convolutional, batch normalization, dropout, and maximum pooling to extract more complex EEG features. Second, it also reduces the dimensionality of EEG features and ensures that some neurons are randomly discarded to prevent overfitting of the detection model. Then, the tiling layer is used to convert the EEG feature vector into 1D data to the fully connected layer. Finally, the result is output by the multi-classifier of the softmax layer.

To improve the detection effectiveness, this study focuses on optimizing the hyperparameters of the 1D-CNN model (Ullah et al., 2018; Yu et al., 2022). The related layers of convolutional, pooling, and fully connected are constructed sequentially as follows.

Each neuron is only associated with a local region of the neuron in the previous layer, which serves to divide the neural network into smaller parts. Then, the EEG features are extracted by the convolutional kernel to reduce the complexity of the network. The convolutional model with *l* layers is characterized as:


(1) $$\begin{array}{l} y_{j}^{l}=f\left(\underset{i\in M_{j}}{\sum }x_{i}^{l-1}\omega_{ij}^{l}+b_{i}^{l}\right),j=1,2,...,M \end{array}$$

Where *l* represents the current layer network; *j* is the previous layer network; $x_{i}^{l-1}$ is the *i*^*th*^ feature mapping of the previous layer; $\omega_{ij}^{l}$ represents the convolution kernel of the *i*^*th*^ and *j*^*th*^ layers; $b_{i}^{l}$ is the bias unit; $y_{j}^{l}$ is the *j*^*th*^ feature mapping of the current layer; and *f*(•) is the activation function.

A pooling layer is generally set behind the convolutional layer to capture the key information. The pooling layers mainly include maximum pooling and average pooling, and the mathematical model of the pooling process is given by


(2) $$\begin{array}{l} y_{j}^{l}=f\left[\text{down}\left(y_{j}^{l-1}\right)+b_{j}^{l}\right] \end{array}$$

Where *down*() is the pooling function.

Each neuron of the fully connected layer is connected to the neuron of the previous layer to extract EEG features. After the stacking of several convolutional and pooling layers, one or more fully connected layers are bridged by a tiling layer, and then the mathematical model of the fully connected layer is calculated as:


(3) $$\begin{array}{l} h\left(x^{l}\right)=f\left(\omega x^{l-1}+b\right) \end{array}$$

Where *x*^*l*−1^ is the input of the fully connected layer, that is, the output of the previous *l*−1 layer, *h*(*x*^*l*^) is the output of the fully connected layer, and ω and *b* are the weight coefficients and bias values of the neurons, respectively.

Finally, the EEG is classified by the full connection layer using the softmax activation function. The final layer reduces the vector of length 178–5. The maximum probability of the corresponding types is obtained, and the different EEG classifications are automatically identified.

#### 2.3.2 The hyperparameters of the 1D-CNN model

The hyperparameters are generally classified into two types, including network structure-related and network training-related (Kolar et al., 2021). Among them, the main hyperparameters related to network structure include the number of convolution kernels—the number of filters, convolutional kernel size—the filter size, number of hidden layers—a layer of neurons between the input and output layers, dropout—random deactivation of a certain percentage of neurons, and activation function—whether a neuron should be activated or not. The other hyperparameters include loss function—a measure of how far the predictions deviate from the true value, batch size—the selection of a sample set to update the weights, the number of iterations—the number of times the entire process is repeated, and learning rate—an adjustment parameter in optimization algorithms.

Hyperparameter selection has a significant impact on the performance of the detection model, which takes more time and requires an enriching experience with manual hyperparameter tuning. However, it is difficult to find the optimal set of hyperparameters through manual experience alone; hyperparameter selection can be quickly searched using intelligent optimization algorithms.

### 2.4 Building the MFO optimization model

The MFO takes the moth position as the optimization problem to be solved using the moth lateral positioning mechanism. The algorithm is good at local development and global searching and is highly robust when solving optimization problems and convergence (Mirjalili, 2015).

In the MFO, the moths and flames are the candidate solutions for the algorithm; the flames are the optimal locations and the moths are the motives that keep moving around the search space. Suppose there are *n* moths $X={\left[x_{1},x_{2},...,x_{n}\right]}^{T}$ and the *i*-th moth is $x_{i}={\left[x_{i,1},x_{i,2},...x_{i,d}\right]}^{T}$, where *d* is the dimension of the optimization parameter. The flame is the best position obtained by the current iteration, then the *i*-th flame is $f_{i}={\left[f_{i,1},f_{i,2},...f_{i,d}\right]}^{T}$.

The MFO is inspired by the behavior of natural moths, and the individual moths iteratively update their position around a flame until the best solution is found. The mathematical description is divided into flame-catching and flame-discarding behavior.

Flame-catching behavior: moths *M*_*i*_ with phototropic behavior in nature move toward the nearest flame *F*_*i*_ to themselves, and the mathematical model of its logarithmic spiral flame-catching trajectory is as follows:


(4) $$\begin{array}{l} S\left(M_{i},F_{i}\right)=D_{i}\cdot {\text{e}}^{bt}\cdot \cos\left(2\pi t\right)+F_{j} \end{array}$$

Where *S*(*M*_*i*_, *F*_*i*_) is the updated moth position, *b* denotes the constant associated with the spiral shape, *t* is a random number in the interval [−1, 1], and *D*_*i*_ = *M*_*i*_ − *F*_*i*_ denotes the distance between the moth *M*_*i*_ and the flame *F*_*i*_.

Flame abandonment behavior: the adaptive mechanism is used to reduce the number of flames, and the mathematical model of flame abandonment operation is as follows:


(5) $$\begin{array}{l} F_{flame}=round\left(N-t*\frac{N-1}{T}\right) \end{array}$$

Where *t* and *T* are the current and maximum iterations, respectively, and *N* is the maximum number of flames.

### 2.5 Hyperparameter of 1D-CNN based on MFO

**Figure 5:**
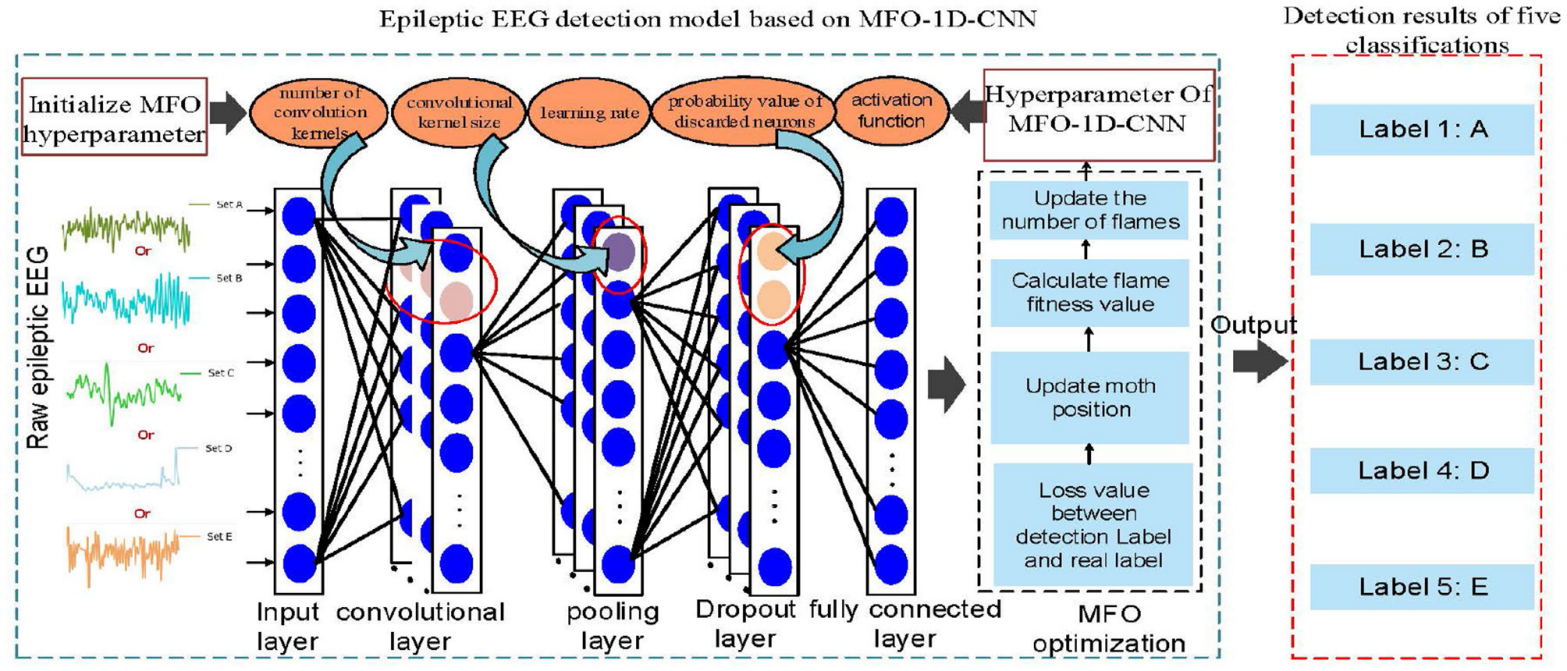
The 1D-CNN hyperparameter tuning based on MFO for detecting EEG classifications.

The hyperparameters of 1D-CNN are still a major obstacle for a small-sample EEG. Setting appropriate hyperparameters not only improves the accuracy of the detection model but also accelerates the speed of model training. Therefore, the MFO automatically optimizes the hyperparameters of the 1D-CNN model, which can improve the feature extraction ability, training efficiency, and detection accuracy. The MFO-1D-CNN model, which combines the MFO with a 1D-CNN model, is proposed, as shown in Figure 5.

The specific process of hyperparameter tuning for the 1D-CNN model using MFO is as follows:

Step 1 initializes the 1D-CNN structure and determines the hyperparameters to be optimized by the MFO. In this work, the 6-dimensional hyperparameter vector is constructed from


(6) $$\begin{array}{l} \lambda =\left[\delta ,s,a,d,\eta ,m\right] \end{array}$$

where λ is the optimized hyperparameter vector, δ is the initial number of kernels, *s* is the size of the kernel, *a* is the activation function, *d* is the probability value of dropped neurons in the Dropout layer, η is the learning rate, and *m* is the pooling window size.

Step 2 adopts the cross-entropy loss function as the objective function and determines the optimization objective functions and restricted conditions; then, the mathematical model is:


(7) $$\begin{array}{l} \min\;L\left(\overset{\wedge }{y},y\right)=-\overset{n}{\underset{j=1}{\sum }}y_{j}\log\overset{\wedge }{y_{j}} \end{array}$$

where *y* is the predicted result of the *j*_*th*_ type, *y*_*j*_ is the true result of the *j*_*th*_ type, *n* is the total number of types, and *L* is the error between the predicted and true value.

The constraints on the mathematical model using the cross-entropy loss function are:


(8) $$\{\begin{array}{l} 40\leq \delta \leq 100,\delta \in N\\ 1\leq s\leq 20,s\in N\\ 0\leq a\leq 2,a\in Z\\ 0\leq d\leq 0.6\\ 0.00001\leq \eta <1\\ 2\leq m\leq 20 \end{array}$$

where *a* = 0, *a* = 1, and *a* = 2 represent the ReLU, sigmoid, and tanh activation functions, respectively.

Step 3 initializes the MFO by setting parameters based on the objective function and constraint conditions and initializes the position of moths in the search space.

Step 4 calculates the distance between the moth and the flame according to the spiral function to update the position of the moth, as shown in Equation (4).

Step 5 calculates the fitness value according to the updated position of the moth and ranks the fitness values in increasing order. The moth position corresponding to the better fitness value is selected as the position of the next generation flame, and the number of flames is updated through the adaptive reduction mechanism, as shown in Equation (5).

Step 6 obtains the position of the optimal flame, which is the current optimal value of each hyperparameter, and the adaptation value of the optimal flame is the current minimum loss value after 1D-CNN training.

Step 7 checks whether the MFO has reached the maximum number of iterations. If not, it returns to step 4. If yes, it sets the optimized hyperparameters to the 1D-CNN network structure and determines the number of epochs, sample batch size, and weight update optimizer.

Step 8 calculates the error between the actual and expected target value by forward propagation based on the EEG dataset and adjusts the weights and biases in the network layer by layer through backpropagation.

Step 9 calculates the validation loss value and accuracy of the current network using the validation dataset at the end of each training cycle. If the validation accuracy is greater than the current optimal value, the currently trained completed network is saved as the optimal model.

Step 10 calculates the number of trainings; if the number is less than or equal to epoch, the process returns to step 8 to continue a new training. If the number is greater than an epoch, this step inputs the test dataset into the saved optimal model and classifies the EEG according to the detection results.

## 3 Experiment setup

### 3.1 Model evaluation and implementation

To achieve a comprehensive evaluation with the optimized 1D-CNN model, the EEG datasets are split at an 80-20 ratio of training and testing data. The tensorflow 2.6.0 in a Python 3.7.13 environment creates the detection models for a Windows workstation equipped with an Intel Xeon Silver 4215*R* CPU, a 64GB memory, and a GTX3080 GPU.

### 3.2 Evaluation metrics

The most important and commonly used parameters for evaluating performance mainly include accuracy, precision, recall, and F1-score (Liu et al., 2020). Among them, accuracy is used to measure the recognition ability of the detection model, precision is used to measure the model's recognition ability to identify an EEG correctly, recall is used to measure the model's ability to find EEGs that are actually positive and predicted to be positive, and F1-score is used as a comprehensive index to comprehensively evaluate a classifier by balancing the effects of accuracy and recall. The specific formulas for evaluating the performance of the detection model are as follows:


(9) $$\begin{array}{l} Accuracy=\frac{TP+TN}{TP+TN+FP+FN}\times 100 \end{array}$$

(10) $$\begin{array}{l} Recall=\frac{TP}{TP+FN}\times 100 \end{array}$$

(11) $$\begin{array}{l} Precision=\frac{TP}{TP+FP}\times 100 \end{array}$$

(12) $$\begin{array}{l} F1-score=\frac{2\times recall\times precision}{recall+precision}\times 100 \end{array}$$

where *TP* (true positive) represents the number of EEG samples correctly classified as positive, *TN* (true negative) represents the number of EEG samples correctly classified as negative, *FP* (false positive) represents the number of EEG samples incorrectly classified as positive, and *FN* (false negative) represents the number of EEG samples incorrectly classified as negative.

## 4 Results and discussion

### 4.1 The effect of different batch sizes on the CNN detection rate

**Figure 6:**
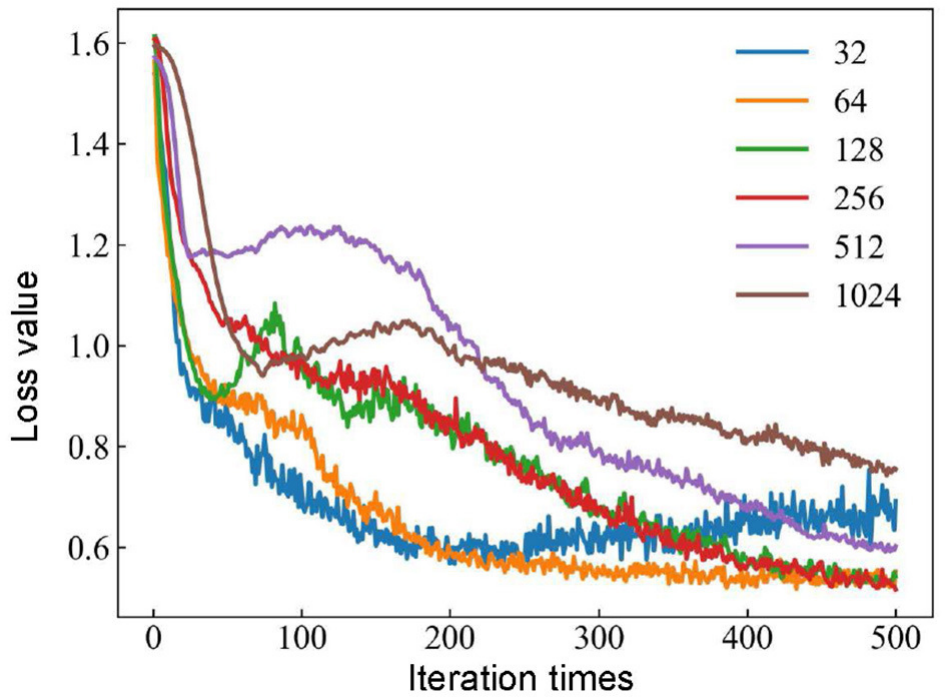
Influence of different batch size conditions for loss curves based on the MFO-1D-CNN model.

The batch size affects the accuracy of the estimate of the error gradient in the process of training neural networks. If the batch size is too small, it takes too much time, and the gradient oscillates severely, which is not conducive to convergence. However, if the batch size is too large, it definitely causes memory overflow. Meanwhile, there is no gradient descent in the gradient direction of different batches, making it easy to fall into the local minimum (Lai et al., 2022). The hyperparameters of the 1D-CNN are optimized based on MFO, and the batch sizes are set to 32, 64, 128, 256, 512, and 1, 024. The new models are evaluated using the detection rate with different batch sizes, and the loss curves under different batch size conditions are shown in Figure 6.

The loss curve tends to stabilize with batch sizes of 32 or 64 when the number of iterations reaches 200. The batch size continues to increase to 128 or 256, and there is a trend of decreasing the loss value. When the batch size is 256, the loss value is the smallest, and when the batch size is further increased to 512 or 1, 024, the trend of the loss value is not decreasing but increasing. The optimal model of the 1D-CNN framework is obtained with a batch size of 256. In addition, the loss value stabilizes at a certain value when the number of iterations reaches 500. Therefore, a good balance between computational efficiency and convergence speed is obtained when the batch size is 256 and the number of iterations reaches 500.

### 4.2 Hyperparameter optimization of the 1D-CNN model

To obtain the optimal solution for improving the detection rate based on a 1D-CNN model, the combination of 6 hyperparameters is considered as the overall dataset, which includes the number of convolution kernels δ, the size of the convolution kernels *s*, the activation function *a*, the probability value of discarded neurons *d*, the learning rate η, and the size of the pooling window *m*. Taking the five-class EEG classification as an example, the neural network hyperparameters are set to achieve the optimal value.

The parameters of the 1D-CNN model can be set to convolutional layers, normalization layers, and dropout layers. The pooling layer uses maximum pooling followed by a tiling layer and a fully connected layer to construct the model of epileptic seizure detection. In addition, the parameters of the 1D-CNN model optimized by PSO are set as follows: the maximum speed of the particle update is 6, the individual learning factor *c*1 is 2, the population learning factor *c*2 is 2, the maximum value of the inertia weight is 0.9, and the minimum value is 0.2. The parameters of the 1D-CNN model optimized by GWO are set as follows: the wolf swarm size is 20, the variable dimension is 6, and the maximum number of iterations is 100. The parameters of the 1D-CNN model optimized by GA are set as follows: the population size is 20, the crossover probability is 1, and the variation probability is 0.01. The parameters of the 1D-CNN model optimized by MFO are set as follows: the moth population size is 20, the variable dimension is 6, and the maximum number of iterations is 30. Through the above optimization parameter settings, the optimal value of the 1D-CNN model is obtained using multiple optimization algorithms, which are iterated 4, 000 times. The different hyperparameters of the 1D-CNN model are shown in Table 1.

**Table 1:** The optimal hyperparameters of 1D-CNN model for the different optimization algorithms.

**Model**	**δ**	***s***	***a***	***d***	**η**	***m***
1D-CNN	40	6	0	3	3	5
PSO-1D-CNN	60	17	0	1	1	2
GA-1D-CNN	80	18	0	2	2	2
GWO-1D-CNN	79	12	0	1	1	2
MFO-1D-CNN	50	10	0	2	2	2

The results of six hyperparameters obtained with different optimization algorithms show obvious differences, and it is very important to select appropriate hyperparameter settings for epileptic seizure detection. Therefore, it is recommended to try multiple optimization algorithms and tune the hyperparameters to find the best training strategy. Therefore, the initial six hyperparameter values are set separately according to the detection results of the 1D-CNN model. In addition, the Adam optimization algorithm is used in the training process, which is an improvement on the random gradient descent algorithm and usually achieves good performance.

### 4.3 The effect of epileptic seizure detection with different optimization algorithms

As each combination of network hyperparameters represents a new model, it takes a lot of time and effort to randomly search for each network parameter. Then, tuning hyperparameters manually is even more time-consuming, and improving efficiency is highly dependent on personal experience. Therefore, this study uses optimization algorithms to find a method that can replace manual parameter adjustment for automatically and effectively finding appropriate network parameters.

**Figure 7:**
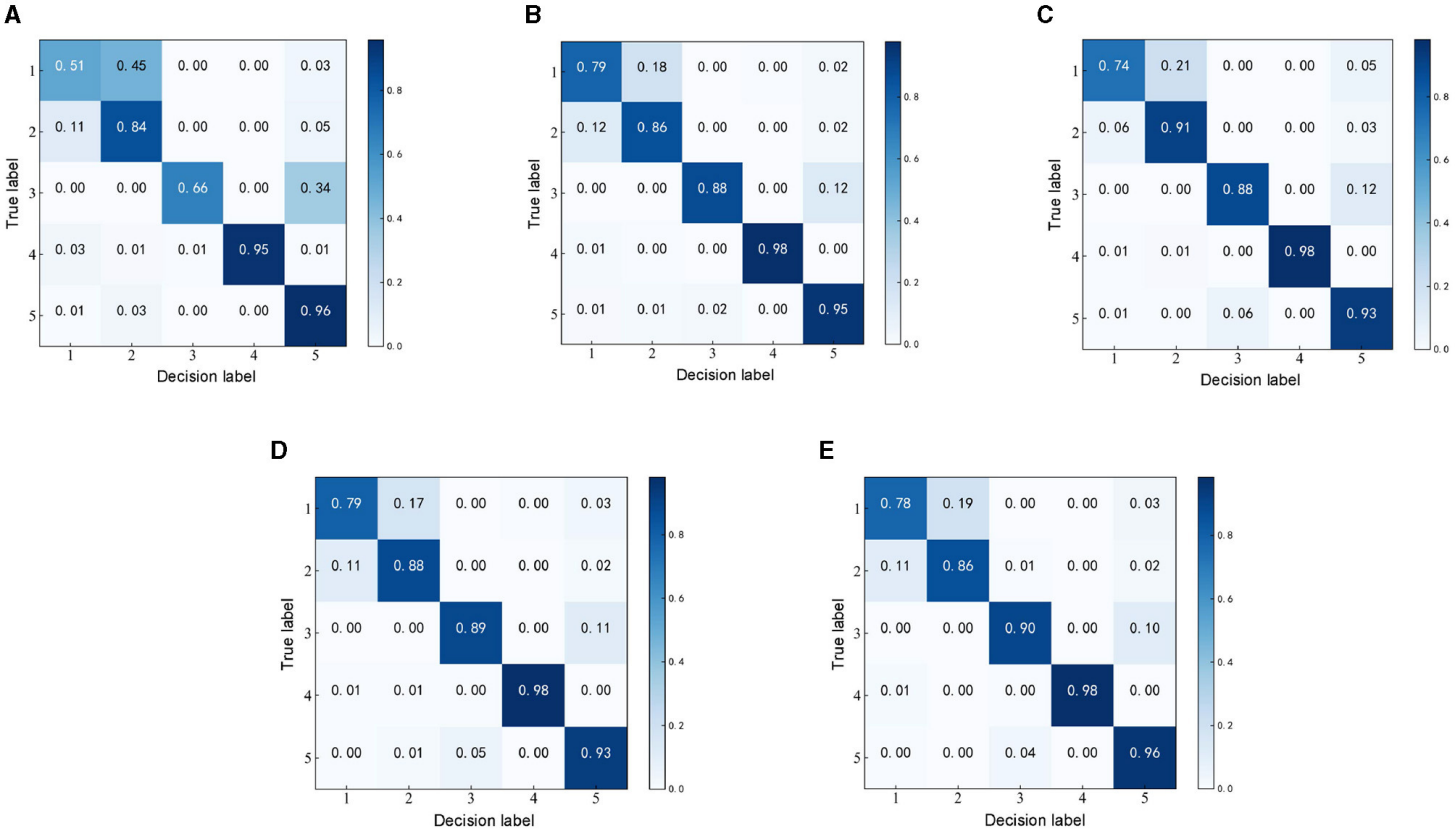
Confusion matrices of the detection models based on different optimization algorithms. **(A)** Confusion matrix of 1D-CNN. **(B)** Confusion matrix of PSO-1D-CNN. **(C)** Confusion matrix of GA-1D-CNN. **(D)** Confusion matrix of GWO-1D-CNN. **(E)** Confusion matrix of MFO-1D-CNN.

To verify the effectiveness of the MFO-1D-CNN model for EEG detection, the representative methods, such as 1D-CNN, PSO-1D-CNN, GA-1D-CNN, and GWO-1D-CNN, are selected to detect seizure using the same EEG dataset and segment with a length of 1 s is selected from the Bonn EEG dataset. As the confusion matrix can be used to summarize the performance of the detection model, the confusion matrices of the five recognition algorithms are compared and analyzed, as shown in Figure 7.

The kappa values calculated by a confusion matrix are used to measure the performance of the detection model. The kappa values corresponding to the 1D-CNN, PSO-1D-CNN, GA-1D-CNN, GWO-1D-CNN, and MFO-1D-CNN models are 0.7300, 0.8717, 0.8600, 0.8697, and 0.8722, respectively. The Kappa level of the optimized 1D-CNN models is almost perfect except for the highly consistent Kappa level of the 1D-CNN model. Although the Kappa levels of all optimization methods perform very similarly, the Kappa values of the MFO-1D-CNN model can still maintain the highest among optimization methods.

**Figure 8:**
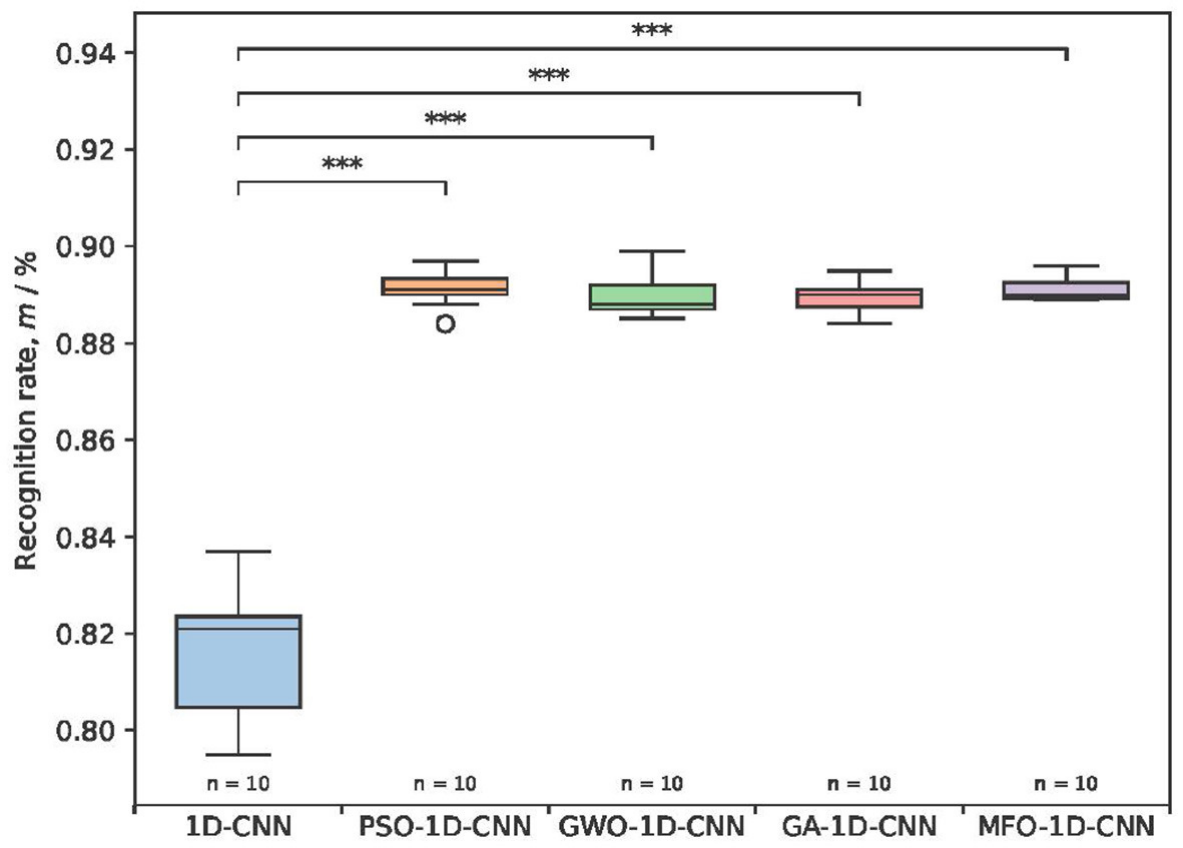
The recognition rates and *p*-values of the detection rates for different optimization models. ***P< 0.001.

To further compare the effectiveness of the differently optimized 1 D-CNN model, the box plots reflect the distribution of the recognition rates and p-values with the different results for the same dataset during each run. To ensure the effectiveness of detection, the result is obtained ten times for the 1D-CNN and four optimized models, and boxes are drawn to show the overall distribution of the detection results, as shown in Figure 8.

The detection rate using the 1D-CNN model ranges from 79.5 to 83.7%, and the recognition rates of the four optimized 1D-CNN models ranged from 88.9 to 89.1%. The recognition rates of the four optimized models are all higher than those before optimization and show significant differences. The distribution of detection rates using the MFO-1D-CNN model is more concentrated, and the time of each detection is 4.10 s, which is slightly better than the 4.16 s for the PSO-1D-CNN model, indicating that the proposed model has a more stable mean recognition rate and a slightly faster detection time. Overall, the comparison with the previous three optimized 1D-CNN models indicates that the MFO-optimized 1D-CNN model is effective at classifying a multi-type EEG.

According to the influence of the 1D-CNN model with different optimization algorithms, the detection results of the proposed model are all better than the other three optimization algorithms based on the Kappa value, recognition rates, and their *P*-values. Therefore, the MFO-1D-CNN model was selected for the subsequent analysis to automatically detect EEG classifications. The hyperparameters of the MFO-1D-CNN model mainly include the number of network layers, the size and number of convolution kernels, and the activation function, as shown in Table 2.

**Table 2:** The MFO-1D-CNN model for epileptic seizure detection.

**Layer number**	**Input shape**	**Layer type**	**Activation size**	**Convolution kernel**	**Output shape**
1	(178, 1)	Conv1D	Relu	10*1	(178, 50)
2	(178, 50)	Batch_Normalization	–	–	(178, 50)
3	(178, 50)	Dropout	–	0.2	(178, 50)
4	(178, 50)	MaxPooling1D	–	2*1	(89, 50)
5	(89, 50)	Conv1D	Relu	10*1	(89, 40)
6	(89, 40)	Batch_Normalization	–	–	(89, 40)
7	(89, 40)	Dropout	–	0.2	(89, 40)
8	(89, 40)	MaxPooling1D	–	2*1	(45, 40)
9	(45, 40)	Conv1D	Relu	10*1	(45, 35)
10	(45, 35)	Batch_Normalization	–	–	(45, 35)
11	(45, 35)	Dropout	–	0.2	(45, 35)
12	(45, 35)	MaxPooling1D	–	2*1	(23, 35)
13	(23, 35)	Conv1D	Relu	10*1	(23, 30)
14	(23, 30)	Batch_Normalization	–	–	(23, 30)
15	(23, 30)	Dropout	–	0.2	(23, 30)
16	(23, 30)	MaxPooling1D	–	2*1	(12, 30)
17	(12, 30)	Conv1D	Relu	10*1	(12, 25)
18	(12, 25)	Batch_Normalization	–	–	(12, 25)
19	(12, 25)	Dropout	–	0.2	(12, 25)
20	(12, 25)	MaxPooling1D	–	2*1	(6, 25)
21	(6, 25)	Conv1D	Relu	10*1	(6, 20)
22	(6, 20)	Batch_Normalization	–	–	(6, 20)
23	(6, 20)	Dropout	–	0.2	(6, 20)
24	(6, 20)	MaxPooling1D	–	2*1	(3, 20)
25	(3, 20)	Flatten	–	–	60
26	60	Dense	Sofmax	5	5

The MFO-optimized 1D-CNN model has 26 network layers, including the input layer, convolutional layer, batch normalization layer, dropout layer, pooling layer, tiling layer, and fully connected layer. Among them, the convolutional layer, the normalization layer of samples, the dropout layer, and the pooling layer are reused several times to improve the EEG recognition rate. According to the results of the proposed model, the hyperparameters are as follows: the initial number of convolutional kernels is (50), the kernel size is (101), the activation function is relu, the probability value is set to (0.2), and the optimizer is Adam. In the last layer of the network, the detection layer is set to (5, 1). The feature vectors are inputted into the detection layer to obtain different probability vectors (5, 1). The parameters of the MFO-1D-CNN model are selected to detect EEG classification so that the proposed model is used for classifying EEG detection in the subsequent study.

### 4.4 Discussion of epileptic seizure detection results

According to the detection need of clinicians, the 4 types of experiments (including 40 sub-experiments) are set up from several different EEG classifications, and these experiments are frequently considered in most studies of epileptic seizure detection. The performance of the MFO-1D-CNN model is analyzed and compared with existing advanced models.

#### 4.4.1 Two-class EEG classification

The seven different two-class classification groups are selected based on the actual needs for detecting an EEG in clinical practice, and the performance of the MFO-1D-CNN model for 23 sub-experiments is calculated based on the proposed model, as shown in Table 3.

**Table 3:** The performance of the proposed model for two-class classification with 10-fold cross-validation.

**Group**	**Class**	**Accuracy**	**Precision**	**Recall**	***F*1-score**
Normal vs. normal	*A* vs. *B*	91.41%	91.56%	91.41%	91.40%
Normal vs. preictal	*A* vs. *C*	98.35%	98.36%	98.35%	98.35%
	*A* vs. *D*	98.65%	98.66%	98.65%	98.65%
	*B* vs. *C*	99.50%	99.50%	99.50%	99.50%
	*B* vs. *D*	99.78%	99.78%	99.78%	99.78%
	*AB* vs. *CD*	98.93%	98.94%	98.93%	98.93%
Normal vs. ictal	*A* vs. *E*	99.96%	99.96%	99.96%	99.96%
	*B* vs. *E*	99.96%	99.96%	99.96%	99.96%
	*AB* vs. *E*	99.90%	99.90%	99.90%	99.90%
Normal vs. preictal and ictal	*AB* vs. *CDE*	99.09%	99.09%	99.09%	99.09%
Preictal vs. preictal	*C* vs. *D*	84.63%	85.08%	84.63%	84.57%
Preictal vs. ictal	*C* vs. *E*	99.78%	99.78%	99.78%	99.78%
	*D* vs.*E*	99.54%	99.55%	99.54%	99.54%
	*CD* vs. *E*	99.70%	99.70%	99.70%	99.70%
Non-ictal vs. ictal	*AC* vs. *E*	99.83%	99.83%	99.83%	99.83%
	*AD* vs. *E*	99.65%	99.65%	99.65%	99.65%
	*BC* vs. *E*	99.84%	99.84%	99.84%	99.84%
	*BD* vs. *E*	99.59%	99.60%	99.59%	99.59%
	*ABC* vs. *E*	99.88%	99.88%	99.88%	99.88%
	*ACD* vs. *E*	99.74%	99.74%	99.74%	99.74%
	*ABD* vs. *E*	99.67%	99.67%	99.67%	99.67%
	*BCD* vs. *E*	99.72%	99.72%	99.72%	99.72%
	*ABCD* vs. *E*	99.77%	99.77%	99.77%	99.77%

The detection rates of the proposed model for 23 small experiments have a high recognition performance of above 90.00%, except for the one in the *C* vs. *D* dataset; the maximum detection rates of an EEG corresponding to normal vs. ictal and non-ictal vs. ictal are even close to 100.00%. This indicates that the MFO-1D-CNN model has excellent classification performance for two-class detection tasks. In addition, the proposed model has good recognition performance for the distribution problems of imbalanced datasets (such as *AB* vs. *CDE*, *CD* vs. *E*, *BCD* vs. *E*, *ABCD* vs. *E*, etc.), and their detection rate reaches ~ 99.00%.

#### 4.4.2 Three-class EEG classification

The five different three-class classification groups including 11 sub-experiments are chosen from five different subsets. The proposed model is used to calculate the evaluation results, as shown in Table 4.

**Table 4:** The performance of the proposed model for three-class classification with 10-fold cross-validation.

**Group**	**Class**	**Accuracy**	**Precision**	**Recall**	**F1-score**
Normal vs. preictal vs. ictal	*A* vs. *C* vs. *E*	98.64%	98.66%	98.64%	98.64%
	*A* vs. *D* vs. *E*	98.77%	98.78%	98.77%	98.77%
	*B* vs. *C* vs. *E*	99.42%	99.42%	99.42%	99.42%
	*B* vs. *D* vs. *E*	98.97%	98.99%	98.97%	98.97%
	*AB* vs. *CD* vs. *E*	98.71%	98.72%	98.71%	98.71%
Normal vs. normal vs. preictal	*A* vs. *B* vs. *C*	92.98%	93.30%	92.98%	93.02%
	*A* vs. *B* vs. *D*	93.39%	93.57%	93.39%	93.41%
Normal vs. normal vs. ictal	*A* vs. *B* vs. *E*	94.17%	94.31%	94.17%	94.18%
Normal vs. preictal vs. preictal	*A* vs. *C* vs. *D*	87.75%	87.72%	87.75%	87.67%
	*B* vs. *C* vs. *D*	89.53%	89.67%	89.53%	89.52%
Preictal vs. preictal vs. ictal	*C* vs. *D* vs. *E*	89.74%	89.94%	89.74%	89.75%

The MFO-1D-CNN model performs well in 11 sub-experiments with an accuracy ranging from 87.75 to 99.42%, and most recognition rates are above 90%. Then, the proposed model achieves an accuracy of 87.75, 89.53, and 89.74% in datasets *A* vs. *C* vs. *D*, *B* vs. *C* vs. *D*, and *C* vs. *D* vs. *E*, respectively. From the above recognition results, the performance of the MFO-optimized 1D-CNN model is still good for three-class EEG classification recognition.

#### 4.4.3 Four-class EEG classification

Similarly, the three different four-class classifications with three sub-experiments are selected. The MFO-1D-CNN model is used to calculate the detection results, as shown in Table 5.

**Table 5:** The performance of the proposed model for four-class classification with 10-fold cross-validation.

**Group**	**Class**	**Accuracy**	**Precision**	**Recall**	**F1-score**
Normal vs. normal vs. preictal vs. preictal	*A* vs. *B* vs. *C* vs. *D*	86.16%	86.56%	86.16%	86.16%
Normal vs. normal vs. preictal vs. ictal	*A* vs. *B* vs. *C* vs. *E*	94.45%	94.68%	94.45%	94.47%
	*A* vs. *B* vs. *D* vs. *E*	94.70%	94.83%	94.70%	94.71%
Normal vs. preictal vs. preictal vs. ictal	*A* vs. *C* vs. *D* vs. *E*	90.86%	90.98%	90.86%	90.84%
	*B* vs. *C* vs. *D* vs. *E*	91.74%	91.93%	91.74%	91.74%

The performance of the proposed model has an accuracy range of 86.16 to 94.70%, and the recognition rates of the last four groups are above 90%, except for the one in the *A* vs.*B* vs.*C* vs.*D* datasets. Generally, the proposed model can adapt to different datasets and has better robustness.

#### 4.4.4 Five-class EEG classification

The five-class classification group consisting of only one sub-experiment is constructed in the *A* vs. *B* vs. *C* vs. *D* vs. *E* datasets. The performance of the proposed model is calculated and evaluated for five-class EEG classification, as shown in Table 6.

**Table 6:** The performance of the proposed model for five-class classification with 10-fold cross-validation.

**Group**	**Class**	**Accuracy**	**Precision**	**Recall**	**F1-score**
Normal vs. normal vs. preictal vs. preictal vs. ictal	*A* vs. *B* vs. *C* vs. *D* vs. *E*	88.34%	88.69%	88.34%	88.32%

The five-class EEG classification is more complex and difficult to classify than the two-class, three-class, and four-class ones. The reason for the difficulty lies in the same category but in different states, such as datasets *A* and *B*, and *C* and *D*. Although there are multiple categories and small differences in the paired datasets, the proposed model still achieves good results, and its average detection rate can still reach 88.34%. This suggests that the MFO-1D-CNN model has excellent recognition performance in five-class EEG classification.

### 4.5 Comparison with existing state-of-the-art detection algorithms

To effectively and reasonably compare and analyze the performance of the proposed model and existing state-of-the-art detection models, the same EEG dataset and segment with a length of 1 s is selected from the Bonn EEG dataset. If there are no studies related to epileptic seizure detection with an EEG segment of 1 s, studies with a similar duration of EEG detection are selected.

First, many researchers have already considered the performance of two-class classification for EEG detection. Subsequently, most of them only focus on the recognition rate of epileptic seizure detection without considering the effect of the duration of the EEG segment, and fewer researchers have studied short-term segments with a duration of 1 s. This study takes 23 combinations of two-class subsets into account to verify the performance of the epileptic seizure detection model using an EEG, and the performance of the proposed model is analyzed and compared with existing advanced detection models, as shown in Table 7.

**Table 7:** Comparison of the performances between the proposed model and existing models on two-class classifications using the same EEG datasets.

**Dataset combination**	**Methods/segment(s)**	**State of the art**	**Acc (%)**	**Our acc (%)**
*A* vs. *B*	CNN + scalogram /23.6 s	Türk and Özerdem, 2019	95.50%	91.41%
*A* vs. *C*	LSTM and SVM/1.12 s	Abbasi et al., 2019	97.78%	98.35%
*A* vs. *D*	1D-LBPall and BayesNet/0.56 s	Kaya et al., 2014	99.50%	98.65%
*B* vs. *C*	CNN + scalogram /23.6 s	Türk and Özerdem, 2019	99.00%	99.50%
*B* vs. *D*	Epileptic-Net /7.87 s	Islam et al., 2022	100.00%	99.78%
*AB* vs. *CD*	LS-SVM/2 s	Sharma et al., 2017	92.50%	98.93%
*A* vs. *E*	1D - CNN/1 s	Zhao et al., 2020	99.52%	99.96%
*B* vs. *E*	1D - CNN/1 s	Zhao et al., 2020	99.11%	99.96%
*AB* vs. *E*	1D - CNN/1 s	Zhao et al., 2020	99.38%	99.90%
*AB* vs. *CDE*	1D-LBPall and BayesNet/0.56 s	Kaya et al., 2014	93.00%	99.09%
*C* vs. *D*	CNN + scalogram /23.6 s	Türk and Özerdem, 2019	80.00%	84.63%
*C* vs. *E*	1D - CNN/1 s	Zhao et al., 2020	99.02%	99.78%
*D* vs. *E*	1D - CNN/1 s	Zhao et al., 2020	97.63%	99.54%
*CD* vs. *E*	1D - CNN/1 s	Zhao et al., 2020	98.03%	99.70%
*AC* vs. *E*	1D - CNN/1 s	Zhao et al., 2020	99.03%	99.83%
*AD* vs. *E*	1D - CNN /1 s	Zhao et al., 2020	98.85%	99.65%
*BC* vs. *E*	1D - CNN /1 s	Zhao et al., 2020	98.68%	99.84%
*BD* vs. *E*	1D - CNN /1 s	Zhao et al., 2020	97.83%	99.59%
*ABC* vs. *E*	1D - CNN /1 s	Zhao et al., 2020	98.89%	99.88%
*ACD* vs. *E*	DWT and kNN /23.6s	Kumar et al., 2012	95.00%	99.74%
*ABD* vs. *E*	1D - CNN /1 s	Zhao et al., 2020	98.56%	99.67%
*BCD* vs. *E*	1D - CNN /1 s	Zhao et al., 2020	98.36%	99.72%
*ABCD* vs. *E*	1D - CNN/1 s	Zhao et al., 2020	98.76%	99.77%

From the above chart, the recognition rates of the proposed model are all above 90%, except for the one in the *C* vs. *D* datasets, which is below 90%. Although the performance of the MFO-1D-CNN model is mostly better than the other existing models on two-class classifications using an EEG, it is worse than the other models in the *A* vs.*B*, *A* vs.*D*, and *B* vs. *D* datasets, and the size of the EEG segment length of the proposed model is far less than the ones of the EEG epoch for the other models. From the comparative analysis of the above research results, it can be observed that the proposed model has a better performance and generalization than the most advanced existing models.

Second, there are also many researchers considering 11 combinations of three-class subsets to verify the performance evaluation based on an EEG. The recognition rate of the proposed model was compared and analyzed with the existing advanced detection model, as shown in Table 8.

**Table 8:** Comparison of the performances between the proposed model and existing models on three-class classifications using the same EEG datasets.

**Dataset combination**	**Methods/segment(s)**	**State of the art**	**Acc (%)**	**Our acc (%)**
*A* vs. *C* vs. *E*	LSTM and SVM/1.12 s	Abbasi et al., 2019	94.81%	98.64%
*A* vs. *D* vs. *E*	1D - CNN/1 s	Zhao et al., 2020	97.04%	98.77%
*B* vs. *C* vs. *E*	1D - CNN /1 s	Zhao et al., 2020	97.91%	99.42%
*B* vs. *D* vs. *E*	1D - CNN/1 s	Zhao et al., 2020	98.06%	98.97%
*AB* vs. *CD* vs. *E*	ResNet-LSTM/1 s	Qiu et al., 2023	98.17%	98.71%
*A* vs. *B* vs. *C*	CNN + scalogram /23.6 s	Türk and Özerdem, 2019	95.00%	92.98%
*A* vs. *B* vs. *D*	CNN + scalogram /23.6 s	Türk and Özerdem, 2019	96.67%	93.39%
*A* vs. *B* vs. *E*	CNN + scalogram /23.6 s	Türk and Özerdem, 2019	95.67%	94.17%
*A* vs. *C* vs. *D*	CNN + scalogram /23.6 s	Türk and Özerdem, 2019	88.00%	87.75%
*B* vs. *C* vs. *D*	CNN + scalogram /23.6 s	Türk and Özerdem, 2019	91.33%	89.53%
*C* vs. *D* vs. *E*	CNN + scalogram /23.6 s	Türk and Özerdem, 2019	89.00%	89.94%

The detection rates of the proposed model are all higher than those of the novel recognition models from the three categories in Abbasi et al. (2019), Zhao et al. (2020), and Qiu et al. (2023). Again, the recognition rate of the proposed model in the *C* vs. *D* vs. *E* datasets is better than that of the detection model in Türk and Özerdem (2019). Although the performance of the proposed model is slightly lower than that of Türk's detection model in the *A* vs. *B* vs. *C*, *A* vs. *B* vs. *D*, *A* vs. *B* vs. *E*, *A* vs.*C* vs. *D*, and *B* vs. *C* vs. *D* datasets, the length of the inputted EEG data of 1 s is much shorter than that of Türk's detection model. From the comparison results, the proposed model is better than that of the known advanced models.

Third, some scholars have also studied five different combinations of four-class subsets to verify the performance of the detection model. Similarly, the performance of the proposed model is further analyzed and compared with existing advanced models, as shown in Table 9.

**Table 9:** Comparison of the performances between the proposed model and existing models on four-class classifications using the same EEG datasets.

**Dataset combination**	**Methods/segment(s)**	**State-of-the-art**	**Acc (%)**	**Our acc (%)**
*A* vs. *B* vs. *C* vs. *D*	Epileptic-Net/7.87 s	Islam et al., 2022	99.91%	86.16%
*A* vs. *B* vs. *C* vs. *E*	Epileptic-Net/7.87 s	Islam et al., 2022	100.00%	94.45%
*A* vs. *B* vs. *D* vs. *E*	Epileptic-Net/7.87 s	Islam et al., 2022	100.00%	94.70%
*A* vs. *C* vs. *D* vs. *E*	CNN + scalogram /23.6 s	Türk and Özerdem, 2019	90.50%	90.86%
*B* vs. *C* vs. *D* vs. *E*	CNN + scalogram /23.6 s	Türk and Özerdem, 2019	91.50%	91.74%

Compared with the detection results in a previous study on four-class classifications (Türk and Özerdem, 2019), the recognition rates of the proposed model are not only slightly higher than those of the previous study for the *A* vs. *C* vs.*D* vs. *E* and *B* vs. *C* vs. *D* vs. *E* datasets but are also obtained through the significantly shorter duration of EEG detection than that of the previous study, with 23.6s. Although the recognition rate of the MFO-optimized 1D-CNN model on the *A* vs. *B* vs. *C* vs. *D*, *A* vs. *B* vs. *C* vs. *E*, and *A* vs. *B* vs. *D* vs. *E* datasets is lower than that of the Epileptic-Net model in the study by Islam et al. (2022), the length of the inputted EEG with 1 s is considerably lower than that of the Epileptic-Net model. In a comprehensive comparison, the proposed model still has advantages with four-class classifications.

Finally, there are relatively few scholars studying the five-class classification because of the difficulty in detecting the five-class EEG. When analyzed and compared with existing advanced models, there is only 1 combination of five-class subsets to verify the performance of the proposed model, as shown in Table 10.

**Table 10:** Comparison of the performances between the proposed model and existing models on five-class classifications using the same EEG datasets.

**Dataset combination**	**Methods/segment(s)**	**State-of-the-art**	**Acc (%)**	**Our acc (%)**
*A* vs. *B* vs.*C* vs. *D* vs.*E*	1D - CNN-LSTM/1 s	Xu et al., 2020	82.00%	88.34%
*A* vs. *B* vs. *C* vs. *D* vs. *E*	1D - CNN/1 s	Zhao et al., 2020	93.55%	88.34%

For the detection results of five categories, a hybrid 1D-CNN and LSTM model is proposed in the study by Xu et al. (2020), and its recognition rate for the *A* vs. *B* vs. *C* vs. *D* vs. *E* datasets is lower than that of the proposed model. The best recognition rate is 93.55% using the 8 optimized models by configuring network parameters manually, the detection rate of which is higher than that of the proposed model in the study by Zhao et al. (2020). Then, considering that configuring the network model requires appropriate professional knowledge and involves tedious processes, the MFO-1D-CNN model has advantages in the two-class, three-class, four-class, and five-class EEG classification problems.

Based on the above analysis, the proposed method can achieve better detection results within 500 iterations. This model is suitable for the 40 different two-class, three-class, four-class, and five-class classifications and has good generalization ability. The MFO-optimized 1D-CNN model not only extends the adaptive adaptability of the model to an EEG but also significantly improves the detection ability of epileptic seizure. Despite achieving good results in epileptic seizure detection, the model still has some shortcomings. First, the proposed model requires a large amount of EEGs. Second, this study does not fully consider the ability to optimize MFO in the optimization process. Finally, the proposed model needs to further reduce the number of neural network layers and neurons to reduce complexity and improve the epileptic seizure detection capability of the MFO-1 D-CNN model.

## 5 Conclusion

This study proposes an automatic epileptic seizure detection model based on 1D-CNN optimized by MFO. The novelty lies in its ability to automatically search for the optimal combination of CNN hyperparameters based on MFO, without manually adjusting the network structure and hyperparameters. This can further improve the effectiveness of the epileptic seizure detection model. The performance of the proposed model in detecting an EEG is experimentally validated with the highest accuracy of 99.96, 99.42, 94.70, and 88.34% in two-class, three-class, four-class, and five-class detection tasks, respectively. In particular, MFO-1D-CNN significantly reduces the time required to detect epileptic seizures. Compared with advanced optimization algorithms such as PSO, GA, and GWO, the proposed model has certain benefits in terms of detection rate and time efficiency. At present, the proposed model is mainly used for the detection of single-channel EEGs. In the future, its ability to predict epileptic seizures will be investigated, and it may be implanted into epileptic seizure detection systems in clinical applications to achieve prediction using EEGs.

## Data availability statement

The original contributions presented in the study are included in the article/supplementary material, further inquiries can be directed to the corresponding authors.

## Ethics statement

Written informed consent was obtained from the individual(s) for the publication of any potentially identifiable images or data included in this article.

## Author contributions

BW: Writing—original draft, Writing—review & editing. XY: Visualization, Writing—original draft. SL: Data curation, Writing—original draft. WW: Software, Writing—original draft. YO: Writing—review & editing. JZ: Project administration, Writing—review & editing. CW: Funding acquisition, Writing—review & editing.

## References

[B1] Abbasi M. U. Rashad A. Basalamah A. Tariq M. (2019). Detection of epilepsy seizures in neo-natal EEG using LSTM architecture. IEEE Access 7, 179074–179085. 10.1109/ACCESS.2019.2959234

[B2] Aliyu I. Lim C. G. (2023). Selection of optimal wavelet features for epileptic EEG signal classification with LSTM. Neural Comp. Appl. 35, 1–21. 10.1007/s00521-020-05666-0

[B3] Andrzejak R. G. Lehnertz K. Mormann F. Rieke C. David P. Elger C. E. (2001). Indications of nonlinear deterministic and finite-dimensional structures in time series of brain electrical activity: dependence on recording region and brain state. Phy. Rev. E 64, 061907. 10.1103/PhysRevE.64.061907 11736210

[B4] Bhattacharyya A. Pachori R. B. Upadhyay A. Acharya U. R. (2017). Tunable-Q wavelet transform based multiscale entropy measure for automated classification of epileptic EEG signals. Appl. Sci. 7, 385. 10.3390/app7040385

[B5] Bongiorni L. Balbinot A. (2020). Evaluation of recurrent neural networks as epileptic seizure predictor. Array 8, 100038. 10.1016/j.array.2020.100038

[B6] Caveness W. Am Lorentz H. Radermecker J. (1964). A proposed international classification of epileptic seizures. Epilepsia 5, 297–306. 10.1111/j.1528-1157.1964.tb03337.x 14273591

[B7] Chetana R. Shubha Rao A. Mahantesh K. (2023). Application of conv-1D and Bi-LSTM to classify and detect epilepsy in EEG Data. Int. J. Adv. Comp. Sci. Appl. 14, 253–261.

[B8] Elsakaan A. A. El-Sehiemy R. A. Kaddah S. S. Elsaid M. I. (2018). An enhanced moth-flame optimizer for solving non-smooth economic dispatch problems with emissions. Energy 157, 1063–1078. 10.1016/j.energy.2018.06.088

[B9] Emami A. Kunii N. Matsuo T. Shinozaki T. Kawai K. Takahashi H. (2019). Seizure detection by convolutional neural network-based analysis of scalp electroencephalography plot images. NeuroImage Clin. 22, 101684. 10.1016/j.nicl.2019.101684 30711680 PMC6357853

[B10] Fatyanosa T. N. Aritsugi M. (2020). “Effects of the number of hyperparameters on the performance of GA-CNN,” in 2020 IEEE/ACM International Conference on Big Data Computing, Applications and Technologies (BDCAT) (Leicester: IEEE), 144–153.

[B11] Hazir E. Erdinler E. S. Koc K. H. (2018). Optimization of CNC cutting parameters using design of experiment (DOE) and desirability function. J. For. Res. 29, 1423–1434. 10.1007/s11676-017-0555-8

[B12] Hernández D. Trujillo L. Flores E. Villanueva O. Romo-Fewell O. (2018). “Detecting epilepsy in eeg signals using time, frequency and time-frequency domain features,” in Computer Science and Engineering—*Theory and Applications* (Springer), 167–182.

[B13] Hoang D.-T. Kang H.-J. (2019). Rolling element bearing fault diagnosis using convolutional neural network and vibration image. Cogn. Syst. Res. 53, 42–50. 10.1016/j.cogsys.2018.03.002

[B14] Irmak E. (2020). Implementation of convolutional neural network approach for COVID-19 disease detection. Physiol. Genom. 52, 590–601. 10.1152/physiolgenomics.00084.2020 33094700 PMC7774002

[B15] Islam M. S. Thapa K. Yang S.-H. (2022). Epileptic-net: an improved epileptic seizure detection system using dense convolutional block with attention network from EEG. Sensors 2, 728. 10.3390/s22030728 35161475 PMC8838843

[B16] Jing J. d'Angremont E. Ebrahim S. Tabaeizadeh M. Ng M. Herlopian A.. (2021). Rapid annotation of seizures and interictal-ictal-injury continuum EEG patterns. J. Neurosci. Methods 347, 108956. 10.1016/j.jneumeth.2020.108956 33099261 PMC7744406

[B17] Kaya Y. Uyar M. Tekin R. Yıldırım S. (2014). 1d-local binary pattern based feature extraction for classification of epileptic EEG signals. Appl. Math. Comput. 243, 209–219. 10.1016/j.amc.2014.05.128

[B18] Khurma R. A. Aljarah I. Sharieh A. (2020). “Rank based moth flame optimisation for feature selection in the medical application,” in 2020 IEEE Congress on Evolutionary Computation (CEC) (Glasgow: IEEE), 1–8.

[B19] Kim S.-H. Geem Z. W. Han G.-T. (2020). Hyperparameter optimization method based on harmony search algorithm to improve performance of 1D CNN human respiration pattern recognition system. Sensors 20, 3697. 10.3390/s20133697 32630344 PMC7374394

[B20] Kolar D. Lisjak D. Pajk M. Gudlin M. (2021). Intelligent fault diagnosis of rotary machinery by convolutional neural network with automatic hyper-parameters tuning using bayesian optimization. Sensors 21, 2411. 10.3390/s21072411 33807427 PMC8036431

[B21] Kong L.-Z. Zhang R.-L. Hu S.-H. Lai J.-B. (2022). Military traumatic brain injury: a challenge straddling neurology and psychiatry. Milit. Med. Res. 9, 2. 10.1186/s40779-021-00363-y 34991734 PMC8740337

[B22] Kumar Y. Dewal M. L. Anand R. S. (2012). Relative wavelet energy and wavelet entropy based epileptic brain signals classification. Biomed. Eng. Lett. 2, 147–157. 10.1007/s13534-012-0066-7

[B23] Kurdthongmee W. (2020). Optimisation of deep neural networks for identification of epileptic abnormalities from electroencephalogram signals. Heliyon 6, e05694. 10.1016/j.heliyon.2020.e05694 33364484 PMC7753124

[B24] Kwasigroch A. Jarzembinski B. Grochowski M. (2018). “Deep CNN based decision support system for detection and assessing the stage of diabetic retinopathy,” in 2018 International Interdisciplinary PhD Workshop (IIPhDW) (Świnouście: IEEE), 111–116.

[B25] Lai H. Zhang L. Zhang S. (2022). Improving network training on resource-constrained devices via habituation normalization. Sensors 22, 9940. 10.3390/s22249940 36560310 PMC9783687

[B26] Lebal A. Moussaoui A. Rezgui A. (2023). Epilepsy-Net: attention-based 1D-inception network model for epilepsy detection using one-channel and multi-channel EEG signals. Multimed. Tools Appl. 82, 17391–17413. 10.1007/s11042-022-13947-0

[B27] Li H. Ding M. Zhang R. Xiu C. (2022). Motor imagery eeg classification algorithm based on cnn-lstm feature fusion network. Biomed. Signal Process. Control 72, 103342. 10.1016/j.bspc.2021.103342

[B28] Liu J. Sun S. Liu Y. Guo J. Li H. Gao Y.. (2020). A novel megnet for classification of high-frequency oscillations in magnetoencephalography of epileptic patients. Complexity 2020, 1–9. 10.1155/2020/9237808

[B29] Lv C. Nian J. Yaru X. Bo S. (2022). “Epilepsy EEG classification and recognition algorithm based on PSO-CNN,” in Second International Conference on Digital Signal and Computer Communications (DSCC 2022), Vol. 12306 (Changchun: SPIE), 320–324.

[B30] Mezzah S. Tari A. (2023). Practical hyperparameters tuning of convolutional neural networks for EEG emotional features classification. Intell. Syst. Appl. 18, 200212. 10.1016/j.iswa.2023.200212

[B31] Mirjalili S. (2015). Moth-flame optimization algorithm: a novel nature-inspired heuristic paradigm. Knowl. Based Syst. 89, 228–249. 10.1016/j.knosys.2015.07.006

[B32] Nikodijevic D. Baneva-Dolnenec N. Petrovska-Cvetkovska D. Caparoska D. (2016). Refractory epilepsy-MRI, EEG and CT scan, a correlative clinical study. Maced. J. Med. Sci. 4, 98. 10.3889/oamjms.2016.029 27275339 PMC4884263

[B33] Qiu X. Yan F. Liu H. (2023). A difference attention ResNet-LSTM network for epileptic seizure detection using EEG signal. Biomed. Signal Process. Control 83, 104652. 10.1016/j.bspc.2023.104652

[B34] Ra J. S. Li T. Li Y. (2023). A novel epileptic seizure prediction method based on synchroextracting transform and 1-dimensional convolutional neural network. Comput. Methods Progr. Biomed. 240, 107678. 10.1016/j.cmpb.2023.107678 37418802

[B35] Sallam A. A. Kabir M. N. Ahmed A. A. Farhan K. Tarek E. (2018). “Epilepsy detection from EEG signals using artificial neural network,” in International Conference on Intelligent Computing & *Optimization* (Cham: Springer), 320–327.

[B36] Sharma M. Pachori R. B. Acharya U. R. (2017). A new approach to characterize epileptic seizures using analytic time-frequency flexible wavelet transform and fractal dimension. Pattern Recognit. Lett. 94, 172–179. 10.1016/j.patrec.2017.03.023

[B37] Shehab M. Abualigah L. Al Hamad H. Alabool H. Alshinwan M. Khasawneh A. M. (2020). Moth-flame optimization algorithm: variants and applications. Neural Comp. Appl. 32, 9859–9884. 10.1007/s00521-019-04570-6

[B38] Thanuja K. Shoba M. Patil K. (2023). Epileptic seizure classification and feature optimization technique using grey wolf algorithm on dynamic datasets. SN Comp. Sci. 4, 311. 10.1007/s42979-023-01741-0

[B39] Tsiouris K. M. Pezoulas V. C. Zervakis M. Konitsiotis S. Koutsouris D. D. Fotiadis D. I. (2018). A long short-term memory deep learning network for the prediction of epileptic seizures using EEG signals. Comput. Biol. Med. 99, 24–37. 10.1016/j.compbiomed.2018.05.019 29807250

[B40] Türk Ö. and Özerdem M. S. (2019). Epilepsy detection by using scalogram based convolutional neural network from EEG signals. Brain Sci. 9, 115. 10.3390/brainsci9050115 31109020 PMC6562774

[B41] Ullah I. Hussain M. Qazi E. Aboalsamh H. (2018). An automated system for epilepsy detection using EEG brain signals based on deep learning approach. Expert Syst. Appl. 107, 61–71. 10.1016/j.eswa.2018.04.021

[B42] Waibel A. Hanazawa T. Hinton G. Shikano K. Lang K. J. (1989). Phoneme recognition using time-delay neural networks. IEEE Trans. Acoust. Speech Signal Process. 37, 328–339. 10.1109/29.2170118282838

[B43] Wang X. Wang X. Liu W. Chang Z. Kärkkäinen T. Cong F. (2021). One dimensional convolutional neural networks for seizure onset detection using long-term scalp and intracranial EEG. Neurocomputing 459, 212–222. 10.1016/j.neucom.2021.06.048

[B44] Wang Y. Zhang H. Zhang G. (2019). cPSO-CNN: An efficient PSO-based algorithm for fine-tuning hyper-parameters of convolutional neural networks. Swarm Evol. Comp. 49, 114–123. 10.1016/j.swevo.2019.06.002

[B45] World Health Organization (2023). Epilepsy. Available online at: https://www.who.int/news-room/fact-sheets/detail/epilepsy/ (accessed August 5, 2023).

[B46] Xu G. Ren T. Chen Y. Che W. (2020). A one-dimensional CNN-LSTM model for epileptic seizure recognition using EEG signal analysis. Front. Neurosci. 14, 578126. 10.3389/fnins.2020.578126 33390878 PMC7772824

[B47] Yu Z. Lu Y. An Q. Chen C. Li Y. Wang Y. (2022). Real-time multiple gesture recognition: application of a lightweight individualized 1D CNN model to an edge computing system. IEEE Transact. Neural Syst. Rehabil. Eng. 30, 990–998. 10.1109/TNSRE.2022.3165858 35394913

[B48] Zawbaa H. M. Emary E. Parv B. Sharawi M. (2016). “Feature selection approach based on moth-flame optimization algorithm,” in 2016 IEEE Congress on Evolutionary Computation (CEC) (Vancouver, BC: IEEE), 4612–4617.

[B49] Zhang H. Liu J. Wang B. Dai J. Lian J. Ke A.. (2022). Motion direction prediction through spike timing based on micro capsnet networks. Science China Technol. Sci. 65, 2763–2775. 10.1007/s11431-022-2072-9

[B50] Zhang T. Chen W. Li M. (2017). Ar based quadratic feature extraction in the VMD domain for the automated seizure detection of EEG using random forest classifier. Biomed. Signal Process. Control 31, 550–559. 10.1016/j.bspc.2016.10.001

[B51] Zhang Y. Yao S. Yang R. Liu X. Qiu W. Han L.. (2022). Epileptic seizure detection based on bidirectional gated recurrent unit network. IEEE Transact. Neural Syst. Rehabil. Eng/ 30, 135–145. 10.1109/TNSRE.2022.3143540 35030083

[B52] Zhao W. Wang W. (2020). SeizureNet: a model for robust detection of epileptic seizures based on convolutional neural network. Cogn. Comp. Syst. 2, 119–124. 10.1049/ccs.2020.0011

[B53] Zhao W. Zhao W. Wang W. Jiang X. Zhang X. Peng Y.. (2020). A novel deep neural network for robust detection of seizures using EEG signals. Comput. Math. Methods Med. 10.1155/2020/9689821 32328157 PMC7166278

